# Use of dual ART in individuals with different HBV serological patterns

**DOI:** 10.1097/COH.0000000000001016

**Published:** 2026-03-26

**Authors:** Thomas Swaine, Sanjay Bhagani

**Affiliations:** a Department of HIV Medicine, Royal Free London NHS Trust; b Division of Infection and Immunity, UCL, London, UK

**Keywords:** hepatitis B, hepatitis B reactivation, HIV, two drug regimens

## Abstract

**Purpose of review:**

Two-drug regimens (2DR) for antiretroviral therapy are increasingly being recommended for people living with HIV to reduce pill burden and reduce short and long-term toxicity. The exclusion of tenofovir and lamivudine or emtricitabine has implications for acquisition of and reactivation of hepatitis B.

**Recent findings:**

A number of case-series, cohort studies and randomized-controlled trials of 2Drs have reported on the risk of HBV acquisition and HBV-reactivation (HBVr). The risk of HBVr appears negligible in those switching to 2DRs containing lamivudine, and overall low (~1%) in those switching to tenofovir and lamivudine/emtricitabine-free therapy. Even remote HBsAg positivity is associated with a significant risk of HBVr.

**Summary:**

For people with HIV switching to 2DRs careful attention needs to be paid to preswitch HBV serological patterns. For those without previous exposure, and absence of HBsAb, vaccination is important. The risk of HBVr of switching to lamivudine-containing regimens is negligible, and low without tenofovir and lamivudine/emtricitabine. Careful monitoring and preswitch counselling is advised.

## INTRODUCTION

Dual ART or two-drug regimens (2DR) have garnered significant interest in contemporary HIV medicine over the last decade. With robust randomized-control trial evidence to demonstrate comparable efficacy to the previous three-drug regimens (3DR) paradigm, the 2DRs dolutegravir/lamivudine (DTG/3TC), dolutegravir/rilpivirine (DTG/RPV) and injectable long-acting cabotegravir/rilpivirine (CAB/RPV-LA) are now recommended as potential first line or alternative and switch regimens for treatment naïve and ART-experienced patients in most clinical guidelines [1,2]. These regimens indeed represent particularly attractive choices for many clinicians and patients; 2DRs can facilitate an alternative to daily oral medication in the form of long-acting intramuscular (CAB/RPV-LA) [3], and by virtue of containing fewer agents can reduce the number of drug-drug interactions and long-term toxicities seen with staple drug in 3DRs. The NRTI “backbone” agent tenofovir is in particular focus here, with both prodrug formulations tenofovir disoproxil fumarate and (TDF) tenofovir alafenamide fumarate (TAF) being associated with significant long-term toxicities (renal impairment and bone demineralization [4,5], and unfavorable metabolic impacts respectively [6]) [7] and thus a move towards “tenofovir-sparing” 2DRs of significant appeal for indefinite antiretroviral therapy.

However, any such move to these “tenofovir-sparing” 2DRs for people with HIV (PWH) must take stock of another virus as well, hepatitis B virus (HBV). Tenofovir remains a first line antiviral treatment for chronic Hepatitis B due to its efficacy and high barrier to resistance for HBV [7], a facet that has no doubt helped cement its prevalence as a key constituent of the majority of antiviral regimens for the last decade given the prevalence of hepatitis B infection world-wide and especially amongst cohorts living with HIV. “Tenofovir-sparing” 2DRs regimens necessarily feature either a greatly weakened (i.e. containing lamivune), or indeed entirely lack anti-HBV activity, with the significance of this varying depending on an individuals’ HBV serological status. Guidance on navigating this has been relatively lacking however with the seminal 2DR RCTs invariably excluding past and current HBV infection [3,8–10], and clinical guidelines subsequently erring for a similarly conservative approach [1,2]. However, recent real-world experience employing 2DRs in clinical practice has yielded greater insight on this topic whilst also highlighting key areas in need of further research. 

**Box 1:**
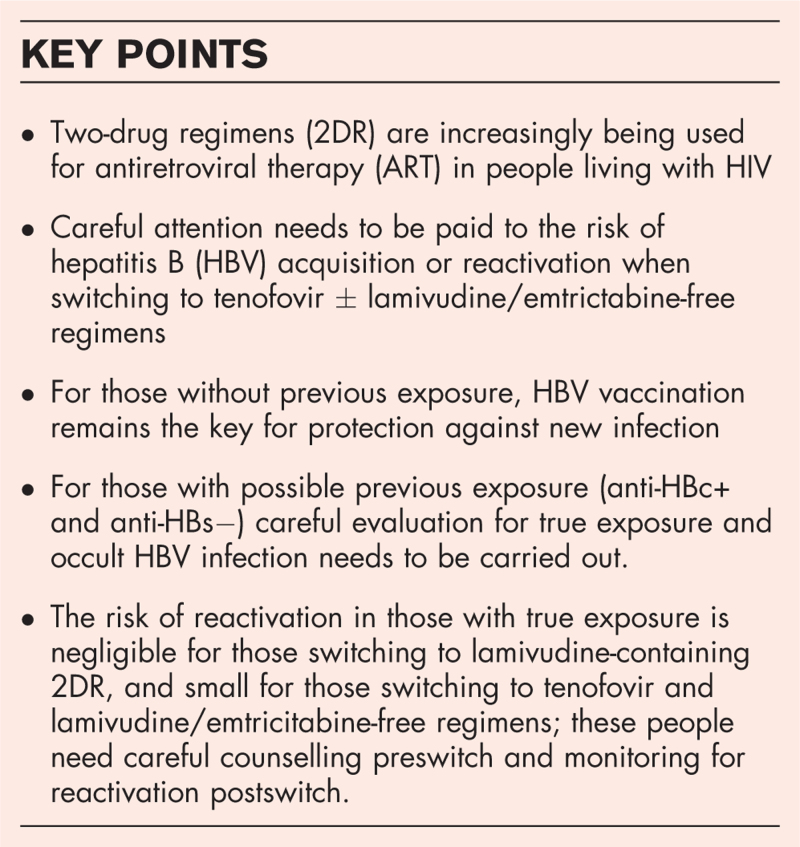
no caption available

## HEPATITIS B SURFACE ANTIGEN NEGATIVE, CORE ANTIBODY NEGATIVE, SURFACE ANTIBODY NEGATIVE INDIVIDUALS (HEPATITIS B SUSCEPTIBLE)

Many sub-groups amongst PWH are at an elevated risk of HBV acquisition due to the shared nature of transmission between the two viruses. Whilst the ubiquitous use of tenofovir containing ART regimens may have had the added benefit of protecting susceptible individuals against HBV infection, its inclusion in regimens was not intended to remove the need for vaccination against Hepatitis B and guidelines consistently recommend vaccination of seronegative individuals [2]. Although HBV vaccination in PWH has been previously plagued by suboptimal responses to conventional vaccine preparations [11], the novel CpG 1080 adjuvanted recombinant vaccine HEPLISAV-B has been demonstrated to consistently achieve protective titers in PWH [12–14] and should alleviate this issue where available for use.

## HEPATITIS B SURFACE ANTIGEN POSITIVE INDIVIDUALS (CHRONIC HEPATITIS B CO-INFECTION)

Understanding and classification of chronic HBV, characterized by positive hepatitis B surface antigen (HBsAg) serology, has evolved significantly in the last 2 decades, and it is now widely recognized to be a complex and dynamic condition featuring several distinct virological, immunological and clinical phases. In HBV mono-infection, not all HBsAg positive individuals are anticipated to receive benefit from antiviral treatment, and criteria of whether and when to stop and start antivirals vary between the various national and international guidelines [7,15]. Conversely, in HIV/HBV co-infection the combination of an accelerated progression to fibrosis and hepatocellular carcinoma compared to mono-infection, and the overwhelming benefits effective ART afford to PWH have rendered this nuance an effective moot point in contemporary practice. ART is universally recommended for all PWH, and in HIV/HBV co-infections only regimens with robust anti-HBV activity (i.e. tenofovir-containing) are recommended, and blanket recommendations against the use of 2DRs in HBsAg positive individuals exist. Indeed, any discontinuation of tenofovir therapy is strongly warned against in co-infection due to the risk of hepatitis flare when tenofovir is withdrawn, although substitution of tenofovir with entecavir alongside fully active ART may be considered where necessary (i.e. absolute contraindication to tenofovir) [1,2].

The question of whether these recommendations need be so ironclad, particularly when considering the fixed-dose single-tablet 2DR, dolutegravir/lamivudine (DTG/3TC), remains unanswered and arguably largely unasked at this venture. As previously alluded to, HBsAg positivity encapsulates a multitude of Hepatitis B disease phases and is well recognized to include individuals with minimally active virus at a negligible increased risk of future hepatic complications; at a virological level it has been demonstrated that detectable HBsAg in serum can be derived from integrated HBV DNA independent of replicating virus [16,17]. Furthermore, though lamivudine (3TC) has been firmly supplanted in current practice, it was previously widely employed for monotherapy of Hepatitis B and continues to be employed for prophylaxis against reactivation for some indications [7,18]. Undoubtedly, in HBV mono-infection the risk of emergent resistance on lamivudine (annualized risk of 22%) [19] coupled with alternative higher genetic barrier agents (tenofovir and entecavir) that entail no additional pill burden and need only be initiated at a higher threshold of disease activity give little reason to challenge this practice, but in co-infection the different calculus at play deserves further scrutiny.

Indeed, several studies have indicated lamivudine only therapies could be safely employed for some risk-stratified co-infected individuals based on e-antigen status or HBV DNA levels [20,21]. Most impactfully, further analysis of the DART trial following up a large cohort in a resource-limited setting found that of the 58 patients with co-infection treated with lamivudine as the sole anti-HBV agent in their ART, a durable suppression of HBV DNA to <48 iu/ml was achieved at almost 5 years in all but one of the patients who had a baseline HBV DNA of a <6 log_10_ IU/ml [22]. Further scoping work is apace currently, with the small-scale study, REFINE-B assessing the acceptability of a reduction from tenofovir/entecavir containing regimens to a 2DR of DTG/3TC in a cohort of 12 co-infected individuals [23], and the results of this and further studies will hopefully inform a more personalized treatment approach for HBsAg positive individuals in the future.

## HEPATITIS B SURFACE ANTIGEN NEGATIVE, ANTI-HBc POSITIVE INDIVIDUALS (RESOLVED HEPATITIS B INFECTION)

The isolated anti-HBc serology is not uncommon (15–30%) in PWH and may represent a myriad of states including occult infection (HBsAg negative, HBV DNA either intermittently or persistently detectable), HBsAg diagnostic escape mutations, a false positive anti-HBc, or truly resolved HBV infection [24–27].

Whilst the presence of anti-HBe may help distinguish between true anti-HBc status and false-positive anti-HBc, and appearance of anti-HBs (anamnestic response) four weeks after a single dose of HBV vaccine may be reassuring in terms of identification of resolved HBV [28], a lack of detectable HBV DNA at a single time-point may not exclude those with occult HBV replication.

Even in resolved infection, characterized by HBsAg negative but anti-HBc positive serology, HBV can pose a risk to individuals due to the virus's capacity to reactivate in the face of a loss of immune control. Hepatitis B reactivations (HBVr) are defined by the re-emergence of HBsAg (seroreversion) and/or HBV viremia and are at risk of progression to significant and potentially fatal hepatitis flares [29]. Notably, the criteria for HBVr based on HBV DNA without HBsAg seroreversion is not consistent in the literature, although a cut off of >100 IU/ml from previously undetectable is typically employed in international guidance [7,30]. Reports of HBVr in patients on 2DRs in recent years have understandably captured attention and heightened concerns about the safety of 2DRs in resolved HBV infection [31^▪^,^32^], but the actual incidence and risk of HBVr in this cohort is murky.

In the realm of mono-infection, the risk of HBVr from a resolved HBV infection is understood to be nigh negligible in the absence of immunosuppression, and representative low-risk therapies including anti-TNF therapy and immune checkpoint inhibitors are estimated to convey a baseline risk of 2 in 1000 and <0.1% respectively [33^▪^]. Notably, whilst historically particular interest had been given to the role Hepatitis B Surface Antibody (anti-HBs) status plays in mitigating risk [34,35], the impact is not judged to be durable by guidelines in most instances, and a more cautious assessment excluding the impact of anti-HBs titers is advised.

For PWH and past HBV infection, extremely varied reports of HBVr risk switching to regimens lacking HBV activity have been made in recent years, ranging to as high 10% risk in the absence of significant immunosuppression in some widely cited work [36]. Such an estimate would put the risk of reactivation in a person with well controlled HIV on par with immunosuppression for solid organ transplant or B-cell depletion [7,30,33^▪^], beggaring biological plausibility. Accordingly, the veracity and generalizability of these estimates require careful scrutiny.

The highest HBVr risk estimate of 10% is derived from subgroup analysis of the randomized control MANET cohort [37]. Undertaken in sub-Saharan Africa MANET compared boosted darunavir monotherapy against a standard of care 3DR containing tenofovir, and amongst the cohort switched to darunavir monotherapy 60 HBsAg negative, anti-HBc positive individuals were identified of whom 6 were reported to have possible HBVr [36], however on closer review these classifications are debatable. Three of the six were found to have a single resolving episode of detectable HBV DNA (<100 iu/ml) without HBsAg seroreversion during the 48-week period, and a further 1 individual had a detectable HBsAg result at a single timepoint without detectable HBV DNA that was not confirmed on follow up testing. Another individual was found to have low level detectable HBV DNA (<250 iu/ml) at two time points without seroreversion, and a final individual had HBsAg seroreversion and low level HBV DNA (<100 iu/ml) without increase in transaminase levels. Notably, this trial was undertaken in a region of high Hepatitis B endemicity, and an incidence of *de novo* hepatitis B acquisition was noted in 6.7% of susceptible individuals during the 48 weeks. Undoubtedly the 10% risk of HBVr reported here is inflated, and any interpretation of potential HBVr events at significant risk of confounding by the ongoing high levels of exposure to HBV evident amongst the study population.

A lower estimated risk of 1.6% is reported in a retrospective review of a large U.S. veterans cohort of 7081 HBsAg negative, anti-HBc positive participants switched to any ART regimen lacking anti-HBV activity prior to 12/31/2022 [38]. However, despite the large sample size there are significant limitations to the data presented in this study owing to the retrospective uncontrolled nature of this analysis. Details on the presence of potentially confounding intercurrent immunosuppression were not available for this analysis, and indeed confirmation that the HBVr cases occurred whilst individuals were still on regimens lacking anti-HBV could only be made in <50% of cases. Furthermore, the definition of HBVr employed for this analysis included any detection of HBV DNA following ART switch without granular data provided on seroreversion rates, the duration that HBV DNA remained detectable for, or correlation with clinical outcomes. One important lesson from the veterans cohort is that the “ever HBsAg +ve” patients (likely representing people with HBsAg loss on treatment) had a higher reported HBVr rate of 17.5–20.7% compared to the 0.4–1.1% rate seen in “never HBsAg” positive patients. Similarly in a recent single-center case series from London [39^▪▪^], two out four patients described with HBVr had documented remote HBsAg loss with tenofovir-based ART prior to 2DR switch, as did the case reported by Adachi *et al.* [32].

Small prospective cohort studies specifically designed to investigate HBVr in PWH provide a significantly more optimistic estimate and may point to a risk profile no different from people not living with HIV (PnLWH) for the majority. Amongst a Spanish cohort of 77 HBsAg negative, anti-HBc positive participants, 20 were switched to 2DRs lacking anti-HBV activity (DTG/RPV or CAP/RPV-LA) and saw no episodes of HBVr over the median 2.1 years of follow up [40]. One participant was noted to have a single episode of transient resolving HBV DNA <100 iu/ml, potentially in keeping with the overreported HBVr rates seen in previous publication. Similarly, analysis of an Italian prospective observational cohort that included 40 HBsAg negative, anti-HBc positive participants switched to 2DRs with no anti-HBV activity demonstrated no hepatitis flares or episodes of HBsAg seroreversion on 2DRs, and indeed also demonstrated no significant increase in transaminase rises compared to HBV-naïve counterparts [41^▪▪^]. Whilst HBV DNA was not routinely measured in this cohort unless transaminase elevations were present, the authors argue that the clinical significance of any potentially undetected “silent” HBVr episodes to be minimal in the absence of more clearly clinically correlated variables (HBsAg seroreversion and transaminases). In the ICONA cohort study of over 500 PWH switching to CAB/RPV-LAI followed up over 12 months [42^▪▪^], 129 of who were anti-HBc+/HBsAg−, there was a single documented case of HBVr (incidence rate of HBV reactivation 6.25 × 1000 PYFU [95% confidence interval (CI) 0.16–34.8] again occurring only in the context of HBsAg loss on ART [43,44].

This is a view potentially supported by experience in the study of HBVr risk in the treatment of HCV, where transient detectable low level HBV DNA was detectable in up to 10% of cases but did not result in seroreversion or hepatitis flares [45], and notably EASL do not specifically mandate HBV DNA testing in addition to HBsAg for HBVr surveillance [7].

Even if an appreciable risk for HBVr does exist for 2DRs without anti-HBV activity, there is convincing evidence that DTG/3TC should circumvent this issue. Amongst the previously mentioned Spanish and Italian studies, an additional 57 and 53 received DTG/3TC during follow up and similarly reported no episodes of HBVr [40,41^▪▪^]. Furthermore, data from large scale DTG/3TC randomized control trials GEMINI-1/-2 [9], STAT [10], TANGO [46], and SALSA [8] supports this. Amongst the trials 76 participants received DTG/3TC who had HBsAg negative, anti-HBc positive serology, and a negative Hepatitis B Surface Antibody (anti-HBs). No explicit episodes of HBVr were reported, and liver safety events data was encouraging [47]. The London case series [39^▪▪^] does describe perhaps the only ever documented case of HBVr on DTG/3TC; this patient, however, had previously lost HBsAg on treatment. An important caveat to consider however is that lamivudine would be a suboptimal choice to mitigate the additional risk any significant immunosuppressive treatment conveys.

Ultimately, we would argue it would seem reasonable to assess the risk of clinically significant HBVr on 2DRs without anti-HBV activity to fall within the realm of <1%, with 2DR containing 3TC very likely to minimize that risk even further and perhaps make it negligible in the absence of a history of HBsAg loss on ART. This low risk of reactivation should be suitable for HBVr surveillance testing easily integrated into standard ART monitoring practices rather than serving as a contra-indication to use of these agents, although careful consideration should be made of the impact any separate immunosuppression may pose.

**FIGURE 1:**
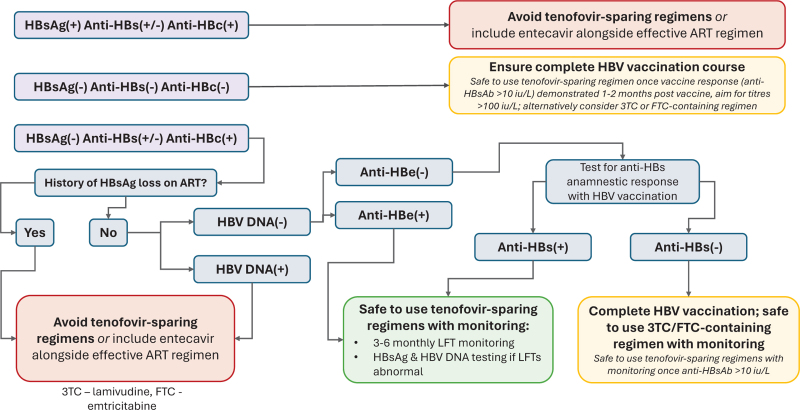
Suggested algorithm for HBV preassessment for the use of tenofovir ± 3TC/FTC –sparing two-drug regimens.

In Fig. 1, we provide an algorithm for the pragmatic preswitch assessment for people starting or switching to tenofovir ± 3TC/FTC sparing regimens.

## CONCLUSION

2DRs are gaining traction as attractive therapy for PWH. Newer less frequent tenofovir-sparing oral regimens (weekly Islatavir/Lenacapavir for example [48]) are on the horizon as are newer injectable therapies.

HBV is becoming an increasing consideration in this regard. Prevention of new HBV infection (in those with no positive serology for HBV) with HBV vaccination is the cornerstone of this endeavor.

The risk of HBVr in those previously exposed to HBV (true anti-HBc+) may be negligible in those without evidence of occult or cryptic HBV replication starting or switching to DTG/3TC.

For tenofovir and lamivudine/emtricitabine-free therapies this risk is low, but not zero. Careful assessment (e.g. Fig. 1) will be necessary; but more importantly patients need to be counselled about the possible risk of HBVr and risks of serious hepatic flares.

Future studies need to address virological and immunological factors associated with this low risk of HBVr and the most cost-effective strategy for postswitch screening.

## Acknowledgements

*None.*

### Financial support and sponsorship

*None.*

### Conflicts of interest

*There are no conflicts of interest.*

## Supplementary Material

## REFERENCES AND RECOMMENDED READING

Papers of particular interest, published within the annual period of review, have been highlighted as:

▪ of special interest
▪▪ of outstanding interest
