# Supplementary material for: Use of dual ART in individuals with different HBV serological patterns
Source: Curr Opin HIV AIDS. 2026 Mar 26;21(3):259–64. doi: 10.1097/COH.0000000000001016 (PMC13048313; doi:10.1097/COH.0000000000001016)
Supplement: Supplementary file 1 [file cohiv-21-259-s001.docx]

**SUPPLEMENTARY MATERIAL**

**New insights on long-term hepatitis B virus responses in HIV-HBV co-infected patients: implications for antiretroviral management in HBV-endemic settings**

**Contents**

|  | **Page** |
| --- | --- |
| Figure 1. Baseline ALT levels in study cohort | 2 |
| Figure 2. Distribution of follow-up time in study cohort | 3 |
| Figure 3. Cross-sectional analysis of HBV DNA levels at the three study time points | 4 |
| Figure 4. HBV DNA level at week 48 versus baseline, stratified by HBsAg status | 5 |
| Figure 5A. HBV DNA level at week 48 versus baseline, stratified by first-line ART regimen: HBsAg-positive patients | 6 |
| Figure 5B. HBV DNA level at week 48 versus baseline, stratified by first-line ART regimen: HBsAg-negative patients | 7 |
| Line listing of HBV DNA, HIV RNA, CD4 count, and ALT values on patients with a sub-optimal HBV DNA response. | 8 |

**Figure 1. Baseline ALT levels in study cohort**

**Figure 2. Distribution of follow-up time in study cohort**

**Figure 3. Cross-sectional analysis of HBV DNA levels at the three study time points**

**Figure 4. HBV DNA level at week 48 versus baseline, stratified by HBeAg status**

HBeAg-positive

HBeAg-negative

**Figure 5A. HBV DNA level at week 48 versus baseline, stratified by first-line ART regimen: HBeAg-positive patients**

**Figure 5B. HBV DNA level at week 48 versus baseline, stratified by first-line ART regimen: HBeAg-negative patients**

**Line listing of HBV DNA, HIV RNA, CD4 count, and ALT values on patients with a sub-optimal HBV DNA response.**

Notes: (1) some Ugandan patients had additional HBV DNA measurements between 4 and 36 weeks (2) As HIV resistance tests were generally not performed, we are not able to distinguish whether increases in HIV RNA levels are due to resistance or non-compliance. Conversely, low HIV RNA values are indicative of good compliance.

**1. Patient with minimal HBV viral load response between baseline and week 48.**

TRIALNO=001, Regimen=TDF

| Week | HBV DNA | HIV RNA | CD4 | ALT |
| --- | --- | --- | --- | --- |
| 0 | 352 |  | 8 | 126 |
| 4 | 649 |  |  | 46 |
| 12 |  |  | 106 | 42 |
| 24 |  |  | 149 | 27 |
| 27 | 40 |  |  |  |
| 36 |  |  | 200 | 20 |
| 48 | 265 |  | 280 | 17 |
| 252 | UD |  | 689 | 13 |
| 276 |  | <400 |  |  |

**2. Patients with HBV viral load rebound after week 48**

TRIALNO=002, Regimen=ABC

| Week | HBV DNA | HIV RNA | CD4 | ALT |
| --- | --- | --- | --- | --- |
| 0 | 96912 | 192148 | 86 | 15 |
| 48 | UD | 238 | 96 | 16 |
| 192 |  | 9522 |  | 13 |
| 216 | 1994 |  | 125 | 26 |
| 228 |  | 12306 | 139 |  |

TRIALNO=003, Regimen=TDF

| Week | HBV DNA | HIV RNA | CD4 | ALT |
| --- | --- | --- | --- | --- |
| 0 | 952 |  | 187 | 36 |
| 48 | BLQ |  | 297 | 23 |
| 192 |  |  | 449 | 35 |
| 252 | 732 |  | 357 | 29 |

TRIALNO=004, Regimen=TDF

| Week | HBV DNA | HIV RNA | CD4 | ALT |
| --- | --- | --- | --- | --- |
| 0 | 27 |  | 40 | 36 |
| 48 | UD |  | 103 | 23 |
| 132 |  |  | 70 | 30 |
| 136 | 298 |  |  |  |
| 144 |  |  | 143 | 27 |

TRIALNO=005, Regimen=TDF

| Week | HBV DNA | HIV RNA | CD4 | ALT |
| --- | --- | --- | --- | --- |
| 0 | 8483312 | 165706 | 112 | 29 |
| 48 | BLQ | >100000 | 173 | 24 |
| 132 | 199 | 22464 | 181 | 19 |

TRIALNO=006, Regimen=TDF

| Week | HBV DNA | HIV RNA | CD4 | ALT |
| --- | --- | --- | --- | --- |
| 0 | 1.43x10^8^ |  | 24 | 39 |
| 48 | UD |  | 73 | 23 |
| 144 | 3485 |  | 4 | 28 |
| 156 |  |  | 306 | 11 |
| 288 |  | 50 |  |  |

TRIALNO=007, Regimen=TDF

| Week | HBV DNA | HIV RNA | CD4 | ALT |
| --- | --- | --- | --- | --- |
| 0 | 3152 |  | 19 | 21 |
| 48 | UD |  | 205 | 39 |
| 276 | 954 |  | 613 | 29 |
| 288 |  | <40 |  |  |

TRIALNO=008, Regimen=TDF

| Week | HBV DNA | HIV RNA | CD4 | ALT |
| --- | --- | --- | --- | --- |
| 0 | 410252 |  | 154 | 46 |
| 48 | BLQ |  | 158 | 23 |
| 240 | 873 |  | 168 | 17 |
| 264 |  | <400 |  |  |

**3. Patients with non-suppressed viral load at week 48 and last time point**

TRIALNO=009, Regimen=TDF

| Week | HBV DNA | HIV RNA | CD4 | ALT |
| --- | --- | --- | --- | --- |
| 0 | 4.97x10^8^ | >750000 | 130 | 43 |
| 48 | 606 | <50 | 204 | 26 |
| 192 |  | <50 |  | 19 |
| 276 | 11974 |  | 218 | 15 |
| 288 |  | <40 |  |  |

TRIALNO=010, Regimen=NVP

| Week | HBV DNA | HIV RNA | CD4 | ALT |
| --- | --- | --- | --- | --- |
| 0 | 126471 | 289478 | 164 | 17 |
| 48 | 147 | <50 | 298 | 18 |
| 216 |  |  |  | 34 |
| 228 |  |  |  | 101 |
| 240 |  | <50 | 576 |  |
| 276 | 1721755 |  |  |  |

TRIALNO=011, Regimen=ABC

| Week | HBV DNA | HIV RNA | CD4 | ALT |
| --- | --- | --- | --- | --- |
| 0 | 4.40x10^8^ | 3165 | 162 | 46 |
| 48 | 598 | <50 | 246 | 9 |
| 96 |  | <50 | 206 |  |
| 228 |  |  |  | 23 |
| 240 | 2.82x10^7^ |  | 351 | 29 |
| 264 |  | <40 |  |  |

TRIALNO=012, Regimen=NVP

| Week | HBV DNA | HIV RNA | CD4 | ALT |
| --- | --- | --- | --- | --- |
| 0 | 7.87x10^7^ | 178977 | 87 | 27 |
| 48 | 4598 | <50 | 104 | 29 |
| 96 |  |  | 82 | 26 |
| 168 | 2.28x10^7^ |  | 103 | 48 |
| 180 |  | <50 |  | 47 |

TRIALNO=013, Regimen=ABC

| Week | HBV DNA | HIV RNA | CD4 | ALT |
| --- | --- | --- | --- | --- |
| 0 | 2.63x10^8^ | 488520 | 131 | 36 |
| 48 | 3397 | 2845 | 276 | 45 |
| 96 |  | 487 | 254 | 14 |
| 192 |  | 234520 | 136 | 23 |
| 204 | 3037024 |  | 221 | 36 |

TRIALNO=014, Regimen=TDF

| Week | HBV DNA | HIV RNA | CD4 | ALT |
| --- | --- | --- | --- | --- |
| 0 | 2.75x10^8^ |  | 47 | 14 |
| 48 | 146 |  | 239 | 24 |
| 96 |  |  | 502 | 27 |
| 216 | 1190 | 47672 | 63 | 19 |

TRIALNO=015, Regimen=NVP

| Week | HBV DNA | HIV RNA | CD4 | ALT |
| --- | --- | --- | --- | --- |
| 0 | 2.58x10^7^ | 425063 | 6 | 30 |
| 48 | 2930 | <50 | 128 | 25 |
| 96 |  |  | 197 | 41 |
| 192 | 7.87x10^7^ |  | 197 | 21 |
| 204 |  | <50 | 276 | 24 |
